# Biventricular Morphology and Function Reference Values Derived From a Large Sample of Healthy Chinese Adults by Magnetic Resonance Imaging

**DOI:** 10.3389/fcvm.2021.697481

**Published:** 2021-07-19

**Authors:** Zhen Zhang, Qiaozhi Ma, Yiyuan Gao, Lizhen Cao, Chengcheng Zhu, Zhiwei Zhao, Jun Zhao, Linan Zeng, Mingwu Lou, Gerald M. Pohost, Kuncheng Li

**Affiliations:** ^1^Post-doctoral Research Center, Department of Radiology, Longgang Central Hospital, Shenzhen Clinical Medical Institute, Guangzhou University of Chinese Medicine, Shenzhen, China; ^2^Department of Radiology, The Third Medical Centre of Chinese PLA General Hospital, Beijing, China; ^3^Department of Radiology, Xuanwu Hospital, Capital Medical University, Beijing, China; ^4^Department of Radiology, University of Washington, Seattle, WA, United States; ^5^Zhouxin Medical Imaging and Health Screening Centre, Xiamen, China; ^6^Keck School of Medicine, University of Southern California, Los Angeles, CA, United States

**Keywords:** cardiovascular magnetic resonance, reference value, left ventricle, right ventricle, Chinese adults

## Abstract

**Background:** Quantification of cardiac structure and function is essential for diagnostic interpretation and clinical decision making. We sought to establish cardiovascular magnetic resonance (CMR) reference values of left and right ventricular (LV and RV) morphology and function based on a large sample of healthy Chinese adults.

**Methods:** Five hundred fifty validated healthy Chinese adults (aged 21–70 years; 323 men) free of hypertension, diabetes, and obesity were included in this study. All the subjects were stratified by gender (men and women) and age decades. On cine CMR, measurements of biventricular end-diastolic, end-systolic, and stroke volumes (EDV, ESV, and SV), ejection fraction (EF), and end-diastolic LV wall thickness (LVWT) and mass (LVM) were obtained.

**Results:** Men had greater LVEDV (111.6 ± 19.8 vs. 94.6 ± 15.6 ml), LVESV (36.5 ± 9.8 vs. 28.2 ± 7.9 ml), LVM (121.1 ± 19.9 vs. 86.1 ± 14.5 g), global end-diastolic LVWT (8.1 ± 1.1 vs. 6.7 ± 1.0 mm), RVEDV (128.0 ± 23.6 vs. 101.7 ± 17.0 ml), and RVESV (53.5 ± 13.7 vs. 36.8 ± 8.9 ml), while women had greater LVEF (67.5 ± 5.4 vs. 70.4 ± 5.7%) and RVEF (58.5 ± 5.2 vs. 64.0 ± 5.3%) (all *p* < 0.001). For both men and women, age was negatively correlated with LVEDV (*r* = −0.31 and *r* = −0.32), LVESV (*r* = −0.37 and *r* = −0.47), RVEDV (*r* = −0.31 and *r* = −0.29), and RVESV (*r* = −0.33 and *r* = −0.44), while it was positively correlated with LVEF (*r* = 0.28 and *r* = 0.43) and RVEF (*r* = 0.28 and *r* = 0.41) (all *p* < 0.001). Aging was associated with increasing global end-diastolic LVWT and LVM/LVEDV in both sexes (all *p* < 0.001). Older age was associated with increasing LVM only in women (*r* = 0.36, *p* < 0.001), not in men (*r* = 0.05, *p* = 0.359).

**Conclusions:** We systematically provide age-, sex-, and body size-specific CMR reference values for biventricular morphology and function based on a large sample of healthy Chinese adults. Biventricular structure and function are significantly associated with age and sex.

## Introduction

The evaluation of left and right ventricular (LV and RV) morphology and function is one of the routine tasks due to its great predicting values in multiple cardiovascular diseases including heart failure, coronary heart disease, cardiomyopathy, and pulmonary diseases (1–5). As a robust non-invasive imaging technique, cardiovascular magnetic resonance (CMR) is playing an important role in a wide range of clinical diagnoses and research due to its excellent accuracy and reproducibility in assessing cardiac structure and function (6).

Normative cardiac reference values are essential to distinguish between healthy and abnormal states in cardiology. Previous studies have confirmed pronounced racial differences in cardiac volumes and function (7–10), emphasizing the necessity of the establishment of ethnic-specific cardiac reference values. Unfortunately, although there are a few CMR studies on normative LV or RV values for healthy Chinese adults (11–13), the sample size is relatively limited. In addition, as a major reference for clinical diagnosis of common cardiovascular diseases, normative values of LV wall thickness (LVWT) for healthy Chinese adults have rarely been reported. Therefore, the present study aims to systematically establish age-, sex-, and body size-specific CMR reference values of LV and RV morphology and systolic functions based on a large sample of healthy Chinese adults with a broad age range.

## Methods

### Study Subjects

Our study population came from the local medical imaging and health screening institution, which aimed at the early detection of various diseases through systemic examinations, with cardiovascular disease screening being one of the key programs. The main screening items included (i) baseline characteristics: age, sex, height, weight, exercise intensity, occupation, and personal and family history, etc., (ii) comprehensive physical examination and electrocardiogram, (iii) laboratory and biochemical tests: routine blood, urine, stool, fasting blood glucose, insulin, lipid, sex hormone, thyroid, liver, kidney function tests, etc., (iv) imaging examination: CMR, echocardiography, chest X-ray film or chest CT, and magnetic resonance examination of the brain, spine, and abdomen. All the examinations for individuals were completed within the same day, and the diagnosis was performed by physicians with more than 10 years of experience. The authors were authorized with full access to all the subject information.

From January 1, 2013, to June 1, 2020, a total of 1,164 consecutive Chinese adults completed the above full-body screening package in the local health screening institution. From this pool of population, subjects who met the following exclusion criteria were excluded: (i) subjects with chief complaint or cardiovascular symptoms, abnormal electrocardiogram (defined as ST-T abnormalities), abnormal cardiac biomarkers including troponin T, pro-brain natriuretic peptide, and C-reactive protein (27.6% of the total screening population underwent the biomarker test); (ii) subjects with abnormal CMR findings of cardiovascular diseases, including ischemic heart diseases (e.g., wall motion abnormalities), hypertrophic cardiomyopathy [defined as end-diastolic left ventricular wall thickness ≥15 mm in any LV segment (14)], congenital heart diseases, valvular heart diseases (defined as observed dephasing jet), and cardiac tumor, etc.; (iii) subjects with known diseases that may affect cardiac morphology and function, such as anemia, hyperthyroidism, gout, and major brain, lung, liver, and kidney diseases; (iv) subjects with hypertension [defined as blood pressure ≥140/90 mmHg or use of antihypertensive medication according to the seventh Joint National Committee recommendation (15)], diabetes [defined as fasting blood glucose (FBG) ≥126 mg/dl or history of hypoglycemic medication], or obesity [defined as body mass index (BMI) ≥28 kg/m^2^ for the Chinese population (16)]; (v) subjects with unqualified images affecting the assessment of LV or RV structure and function, such as artifacts and incomplete or suboptimal presentation of ventricular contours. All the examinations for individuals were completed within the same day. Systolic and diastolic blood pressures (SBP and DBP) were measured at rest according to standard procedure. The present study was approved by the local institutional review board, and the requirement for subject consent was waived by the Chinese Ethics Committee (Reference Number: ChiECRCT20190198).

### Cardiovascular Magnetic Resonance Imaging

CMR studies were performed on 1.5T magnetic resonance scanners (Signa HDxt, General Electric Medical Systems, Waukesha, WI, USA, and Magnetom Essenza, Siemens, Erlangen, Germany) with a 16-channel phased-array surface coil. The subjects were in the supine position, and balanced steady-state free precession (SSFP) sequences were used to acquire cine images in held end expiration. Electrocardiogram-triggered continuos short-axis stack, parallel to the atrioventricular groove, covering the whole left and right ventricle (10–15 slices) was acquired at one slice per breath hold. In addition, long-axis cines (horizontal long axis, vertical long axis, and LV outflow tract) were also obtained. All the individuals were examined under the sinus rhythm. The scanning time for each individual is about 20–35 min.

The parameters of sequence for Signa HDxt, General Electric Medical Systems are as follows: repetition time = 4 ms, echo time = 1.75 ms, flip angle = 60°, field of view = 310 × 310 mm, matrix size = 224 × 224, slice thickness = 8 mm, slice gap = 1 mm, and 30 phases per cardiac cycle. The scanning parameters for Magnetom Essenza, Siemens, are as follows: repetition time = 4.38 ms, echo time = 1.37 ms, flip angle = 60°, field of view = 275 × 340 mm, matrix size = 224 × 256, slice thickness = 8 mm, slice gap = 0 mm, and 30 phases per cardiac cycle.

### Image Analysis

Image analysis was performed using commercial software (cvi42® version 5.6.2, Circle Cardiovascular Imaging, Canada). Images were magnified to 250%. All the measurements were completed by an experienced reviewer (the first author, more than 5 years experience) blinded to all the personal information (including age, gender, and height, etc.).

**Figure 1:**
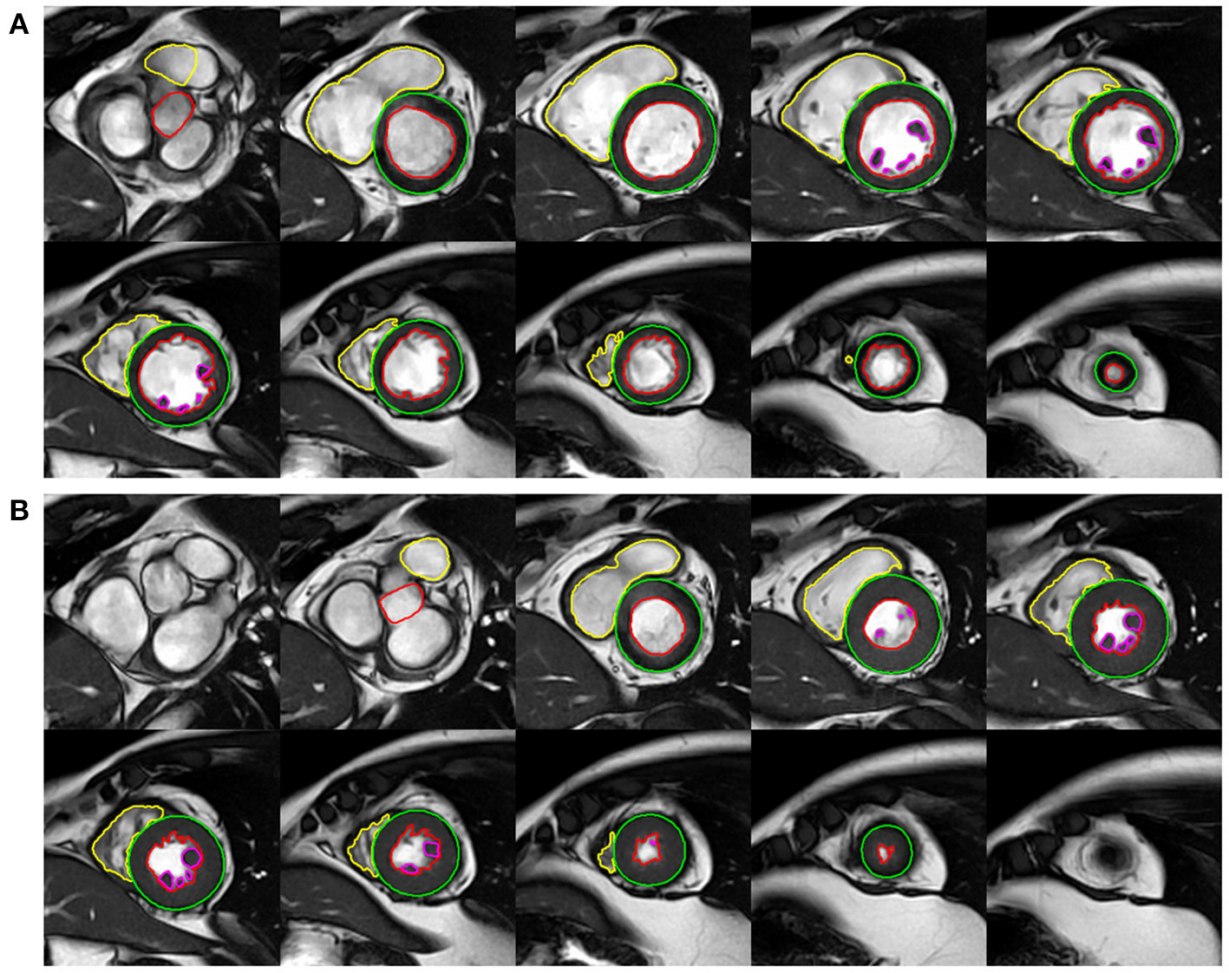
Example of left ventricular (LV) and right ventricular (RV) functional analysis on the short-axis cine images of end-diastole **(A)** and end-systole **(B)**.

The frames with the smallest blood pool by visual assessment in the middle of the ventricular cavity were defined as the end systole and the largest as the end diastole. As for the LV, the most basal section with at least 50% surrounding myocardium was regarded as the base. The most apical section with visible cavity was considered as the apex. On the short-axis cine imaging, the LV endocardial borders were traced by semi-automatic threshold-based segmentation method with manual correction if necessary. The LV epicardial borders were manually traced using the point-and-click method. Trabeculae and papillary muscles (TPM) were excluded from the LV blood pool and included in the myocardium. For the RV, the phases of end diastole and end systole were consistent with the definition of the LV. From the inflow tract, the slice in which the surrounding muscle was thin and not trabeculated, suggestive of the right atrium, were excluded from the RV volumes. In order to improve reproducibility, TPM was included in the RV blood pool according to the recent standards for image interpretation and post-processing (17). The outflow tract to the level of the aortic or pulmonary valve cusps was carefully included in the ventricular volumes by checking the matching long-axis planes. At the end of diastole, the anterior and inferior insertion of the right ventricle was used to define the segments according to the American Heart Association (AHA) 17-segment model (18) (Figure 1).

Biventricular end-diastolic volume (EDV), end-systolic volume (ESV), stroke volume (SV), and ejection fraction (EF) were obtained. The LV mass (LVM) was obtained from the end-diastolic frames. Segmental LVWTs at the end diastole according to the AHA 17-segment model (except for apical cap) were obtained automatically by software on short-axis images. Global end-diastolic LVWT was defined as the average of the segmental wall thickness at end diastole. The mean end-diastolic LVWT at the base, mid-cavity, and apex of the left ventricle were calculated according to the AHA model. Heart rate (HR) was measured at the time of the CMR scan. Cardiac output (CO) was obtained by multiplying SV and HR. Parameters of biventricular structure and function were indexed to body surface area (BSA). BSA was calculated using the Mosteller formula: BSA (m^2^) = [height (cm) × weight (kg)/3,600]^1/2^. The LVM/LVEDV ratio was calculated to assess LV concentricity.

### Intra-observer and Inter-observer Reproducibility

To assess the inter-observer reliability of the LV and RV measurements (biventricular EDV, ESV, EF, and LVM), 50 datasets were randomly selected from the subjects and were measured by two independent observers (both have more than 5 years of experience). To assess the intra-observer reliability, the datasets were measured by an observer for the second time using the same method with a time interval of more than a month.

### Statistical Analysis

All statistical analyses were performed with SPSS (version 25.0, IBM SPSS Inc., Chicago, IL, USA) and GraphPad Prism (version 7.0, La Jolla, CA, USA). All the data were tested by the Shapiro–Wilk-test and were confirmed as normal distribution. The demographic parameters, as well as the measurements of biventricular structure and function, are presented as “mean ± standard deviation.” The gender differences in the demographic characteristics and biventricular measurements were examined using Student's *t-*test. Associations of age with biventricular parameters (EDV, ESV, EF), global end-diastolic LVWT, and LVM were assessed using simple linear regressions. Intra- and inter-observer variability were analyzed using Bland–Altman analysis.

Differences were regarded as statistically significant at *p* < 0.05. All *p*-values were two-sided.

## Results

### Population Characteristics

In the total population of 1,164, a total of 614 subjects were excluded, leaving 550 healthy adults (323 men, 43.1 ± 11.6 years). There was no significant gender difference in age (*p* = 0.660) and HR (*p* = 0.077). Height, body weight, BMI, BSA, SBP, DBP, and FBG were all greater in men (all *p* < 0.001; Table 1).

**Table 1:** Demographic characteristics of the studied population.

	**All (*n* = 550)**	**Men (*n* = 323)**	**Women (*n* = 227)**	***p*-value**
Age (years)	43.1 ± 11.6	43.0 ± 11.1	43.4 ± 12.2	0.660
Height (cm)	167.0 ± 8.1	171.6 ± 6.1	160.4 ± 5.8	<0.001
Body weight (kg)	63.8 ± 10.8	69.5 ± 9.1	55.6 ± 7.1	<0.001
BMI (kg/m^2^)	22.8 ± 2.8	23.6 ± 2.5	21.6 ± 2.6	<0.001
BSA (m^2^)	1.72 ± 0.18	1.82 ± 0.14	1.57 ± 0.11	<0.001
HR (bpm)	66.9 ± 9.6	66.3 ± 9.8	67.8 ± 9.2	0.077
SBP (mm Hg)	114.3 ± 12.9	117.3 ± 10.8	110.0 ± 14.4	<0.001
DBP (mm Hg)	70.4 ± 9.3	72.7 ± 8.7	67.2 ± 9.2	<0.001
FBG (mmol/L)	5.18 ± 0.44	5.24 ± 0.44	5.09 ± 0.43	<0.001

*Data are presented as means ± standard deviation. p-Value is for t-test between genders*.

*BMI, body mass index; BSA, body surface area; HR, heart rate; SBP, systolic blood pressure; DBP, diastolic blood pressure; FBG, fasting blood glucose*.

### Gender Differences in Biventricular Structure and Function

**Figure 2:**
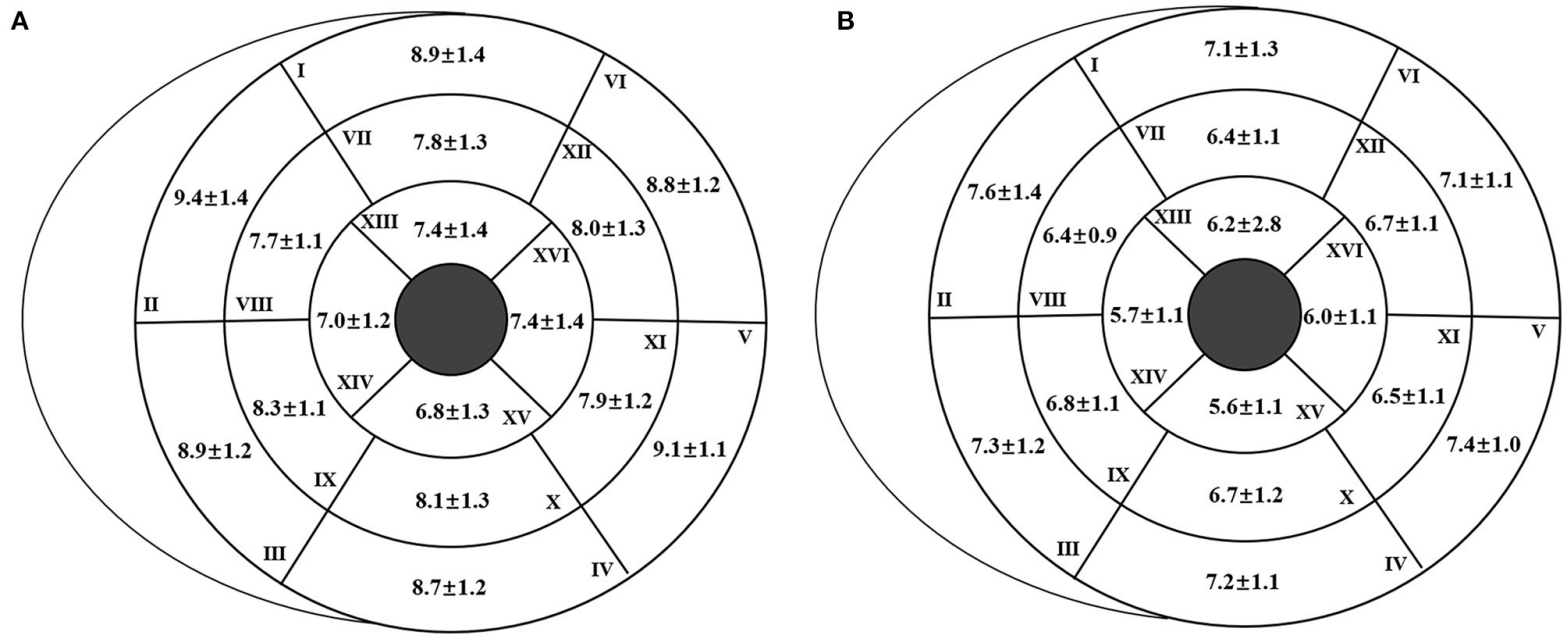
Segmental end-diastolic left ventricular wall thickness (LVWT) [according to the American Heart Association (AHA) model] for men **(A)** and women **(B)**. The end-diastolic LVWT is presented as “mean ± standard deviation.”

The reference values of biventricular structural and functional parameters for both sexes are shown in Table 2. Normal values of segmental end-diastolic LVWTs (according to the AHA model) in men and women are shown in Figure 2. Biventricular EDV, ESV, and SV were all greater in men, while LVEF and RVEF were both greater in women (all *p* < 0.001). After normalized by BSA, all the LV and RV volume parameters were still greater in men than those in women (all *p* < 0.001), except for LVEDV, LVSV, and RVSV (*p* = 0.152, *p* = 0.114, and *p* = 0.574, respectively). Besides, both absolute and indexed LVM and global end-diastolic LVWT were all greater in men (all *p* < 0.001).

**Table 2:** Absolute and indexed left ventricular (LV) and right ventricular (RV) parameters for men and women.

	**All (*n* = 550)**	**Men (*n* = 323)**	**Women (*n* = 227)**	***p*-value**
**Absolute LV values**				
LVEDV (ml)	104.6 ± 20.0	111.6 ± 19.8	94.6 ± 15.6	<0.001
LVESV (ml)	33.1 ± 9.9	36.5 ± 9.8	28.2 ± 7.9	<0.001
LVSV (ml)	71.5 ± 13.2	75.2 ± 13.3	66.3 ± 11.0	<0.001
LVEF (%)	68.7 ± 5.7	67.5 ± 5.4	70.4 ± 5.7	<0.001
LVCO (L/min)	4.8 ± 1.0	4.9 ± 1.0	4.5 ± 0.9	<0.001
LVM (g)	106.6 ± 24.9	121.1 ± 19.9	86.1 ± 14.5	<0.001
Global end-diastolic LVWT (mm)	7.5 ± 1.3	8.1 ± 1.1	6.7 ± 1.0	<0.001
**Indexed LV values**				
LVEDV/BSA (ml/m^2^)	60.9 ± 9.7	61.4 ± 10.1	60.2 ± 9.2	0.152
LVESV/BSA (ml/m^2^)	19.2 ± 5.2	20.1 ± 5.2	18.0 ± 5.0	<0.001
LVSV/BSA (ml/m^2^)	41.7 ± 6.6	41.3 ± 6.8	42.2 ± 6.3	0.114
LVM/BSA (g/m^2^)	61.6 ± 10.3	66.5 ± 9.2	54.7 ± 7.4	<0.001
Global end-diastolic LVWT/BSA (mm/m^2^)	4.4 ± 0.6	4.5 ± 0.6	4.2 ± 0.6	<0.001
**Absolute RV values**				
RVEDV (ml)	117.2 ± 24.8	128.0 ± 23.6	101.7 ± 17.0	<0.001
RVESV (ml)	46.6 ± 14.5	53.5 ± 13.7	36.8 ± 8.9	<0.001
RVSV (ml)	70.6 ± 13.2	74.6 ± 13.2	64.9 ± 11.1	<0.001
RVEF (%)	60.8 ± 5.9	58.5 ± 5.2	64.0 ± 5.3	<0.001
RVCO (L/min)	4.7 ± 1.0	4.9 ± 1.0	4.4 ± 0.9	<0.001
**Indexed RV values**				
RVEDV/BSA (ml/m^2^)	68.0 ± 11.5	70.4 ± 11.6	64.5 ± 10.6	<0.001
RVESV/BSA (ml/m^2^)	26.9 ± 6.9	29.4 ± 6.8	23.4 ± 5.5	<0.001
RVSV/BSA (ml/m^2^)	41.1 ± 6.6	41.0 ± 6.9	41.3 ± 6.3	0.574

*p-value is for t-test between genders*.

*BSA, body surface area; EDV, end-diastolic volume; ESV, end-systolic volume; SV, stroke volume; EF, ejection fraction; CO, cardiac output; LVM, left ventricular mass; LVWT, left ventricular wall thickness*.

### Correlation Between Age and Biventricular Structure and Function

**Figure 3:**
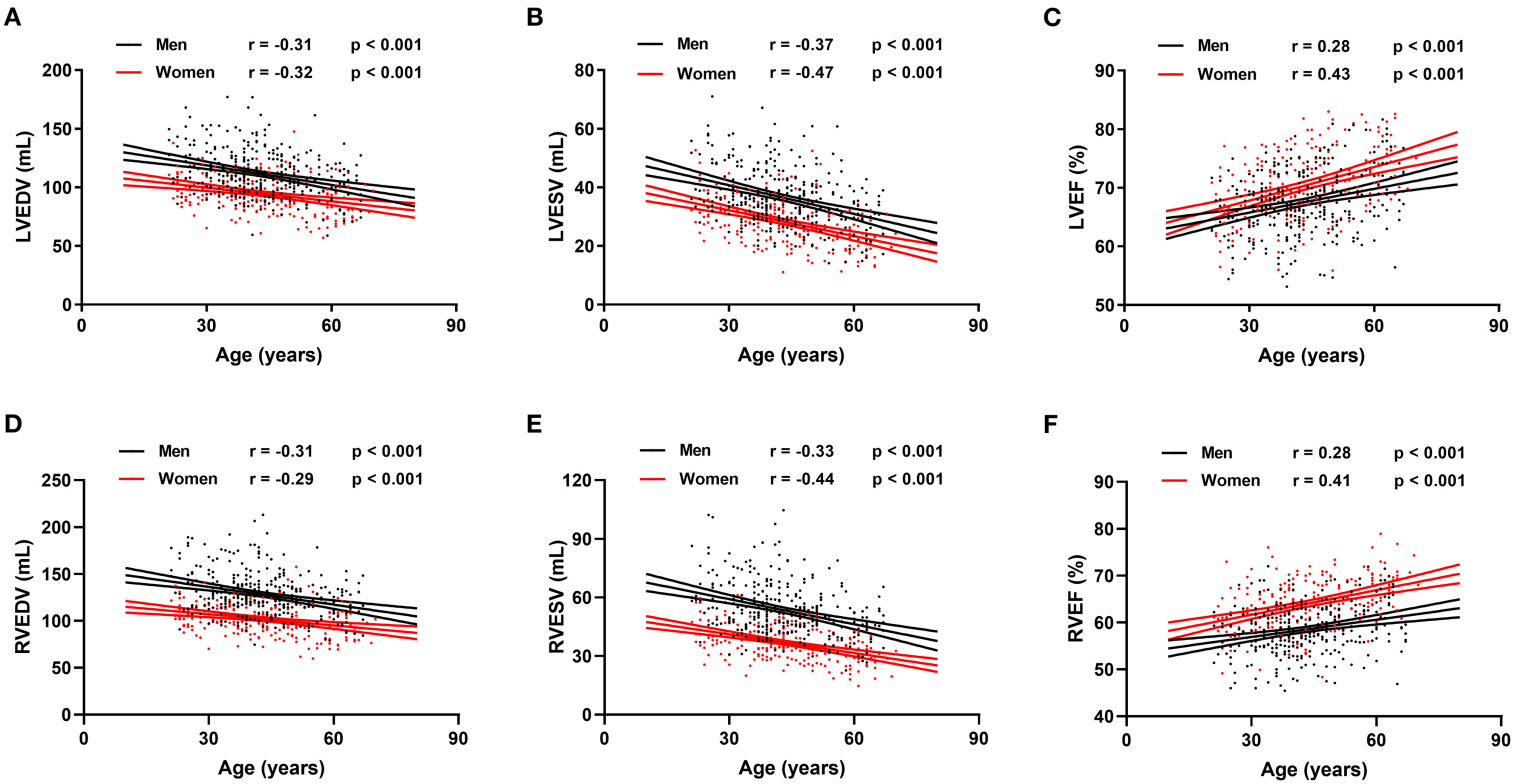
Correlation between biventricular end-diastolic volume (EDV), end stroke volume (ESV), ejection fraction (EF), and age in men and women. Linear regressions with 95% confidence intervals for left ventricular EDV (LVEDV) **(A)**, left ventricular ESV (LVESV) **(B)**, left ventricular EF (LVEF) **(C)**, right ventricular EDV (RVEDV) **(D)**, right ventricular ESV (RVESV) **(E)**, and right ventricular EF (RVEF) **(F)**.

**Figure 4:**
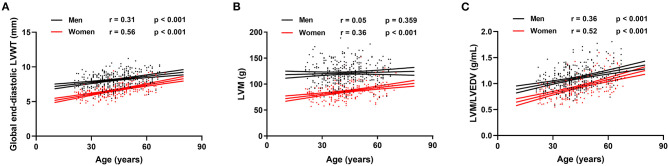
Correlation between global end-diastolic LVWT, left ventricular mass (LVM), LVM/LVEDV, and age for men and women. Linear regressions with 95% confidence intervals for global end-diastolic LVWT **(A)**, LVM **(B)**, and LVM/LVEDV **(C)**.

Reference values of biventricular structure and function stratified by sex and age deciles are shown in Tables 3–8. Overall, the sample proportions of the corresponding age groups in both sexes were not much different. Specifically, the sample proportions of 21–30, 31–40, 41–50, 51–60, and 61–70 age groups are 12.7, 33.4, 29.4, 15.5, and 9.0% in men, and 17.7, 26.4, 26.9, 17.2, and 11.9% for women, respectively. For both sexes, linear regression showed that age was negatively correlated with LVEDV (*r* = −0.31 and *r* = −0.32, both *p* < 0.001), LVESV (*r* = −0.37 and *r* = −0.47, both *p* < 0.001), RVEDV (*r* = −0.31 and *r* = −0.29, both *p* < 0.001), and RVESV (*r* = −0.33 and *r* = −0.44, both *p* < 0.001). In men and women, age was positively correlated with LVEF (*r* = 0.28 and *r* = 0.43, both *p* < 0.001) and RVEF (*r* = 0.28 and *r* = 0.41, both *p* < 0.001) (Figure 3). Moreover, aging was associated with increasing global end-diastolic LVWT in both sexes (*r* = 0.31 and *r* = 0.56, both *p* < 0.001), while it was associated with increasing LVM only in women (*r* = 0.36, *p* < 0.001), not in men (*r* = 0.05, *p* = 0.359). Compared with men, there was a greater age-associated increase in LVM/LVEDV ratio in women (*r* = 0.36 and *r* = 0.52 for men and women, both *p* < 0.001) (Figure 4).

**Table 3:** Absolute and indexed LV parameters by age decile for men (*n* = 323).

	**21–30 (*n* = 41)**	**31–40 (*n* = 108)**	**41–50 (*n* = 95)**	**51–60 (*n* = 50)**	**61–70 (*n* = 29)**
**Absolute values**					
LVEDV (ml)	122.4 ± 20.8	113.3 ± 18.1	111.2 ± 20.2	104.9 ± 17.6	99.7 ± 17.9
LVESV (ml)	43.2 ± 10.7	37.8 ± 8.7	36.0 ± 9.1	33.8 ± 9.5	28.6 ± 7.7
LVSV (ml)	79.2 ± 13.4	75.4 ± 12.8	76.2 ± 14.5	71.0 ± 10.4	71.1 ± 13.5
LVEF (%)	64.9 ± 5.0	66.7 ± 4.9	68.0 ± 5.3	68.2 ± 5.3	71.4 ± 5.8
LVCO (L/min)	5.5 ± 1.3	4.9 ± 1.0	4.9 ± 0.9	4.7 ± 0.9	4.7 ± 1.0
LVM (g)	122.2 ± 21.6	118.2 ± 19.4	122.9 ± 21.0	122.6 ± 18.0	121.9 ± 19.3
**Indexed values**					
LVEDV/BSA (ml/m^2^)	67.5 ± 9.3	62.3 ± 9.2	61.4 ± 10.2	57.3 ± 9.4	56.5 ± 9.9
LVESV/BSA (ml/m^2^)	23.8 ± 5.2	20.8 ± 4.6	19.7 ± 4.8	18.5 ± 5.0	16.3 ± 4.7
LVSV/BSA (ml/m^2^)	43.7 ± 6.3	41.5 ± 6.6	41.7 ± 7.4	38.9 ± 5.7	40.2 ± 7.0
LVM/BSA (g/m^2^)	67.1 ± 8.4	64.9 ± 9.2	67.2 ± 9.9	66.9 ± 8.2	68.8 ± 8.9

*Abbreviations as in Table 2*.

**Table 4:** Absolute and indexed LV parameters by age decile for women (*n* = 227).

	**21–30 (*n* = 40)**	**31–40 (*n* = 60)**	**41–50 (*n* = 61)**	**51–60 (*n* = 39)**	**61–70 (*n* = 27)**
**Absolute values**					
LVEDV (ml)	99.9± 14.6	97.4 ± 12.5	95.0 ± 14.2	89.3 ± 19.8	86.9 ± 15.4
LVESV (ml)	33.5 ± 7.3	29.5 ± 6.2	28.1 ± 7.9	25.3 ± 7.9	22.1 ± 6.1
LVSV (ml)	66.5 ± 10.7	67.8 ± 9.3	66.9 ± 10.4	64.0 ± 14.4	64.8 ± 11.2
LVEF (%)	66.5 ± 4.9	69.8 ± 4.9	70.6 ± 6.1	71.8 ± 5.3	74.8 ± 4.5
LVCO (L/min)	4.6 ± 0.9	4.6 ± 0.7	4.4 ± 0.9	4.3 ± 1.0	4.4 ± 0.8
LVM (g)	79.7 ± 9.1	82.1 ± 13.5	86.6 ± 13.6	91.9 ± 16.5	94.7 ± 15.3
**Indexed values**					
LVEDV/BSA (ml/m^2^)	64.9 ± 7.0	62.7 ± 7.7	59.8 ± 8.6	55.8 ± 10.2	54.9 ± 9.6
LVESV/BSA (ml/m^2^)	21.8 ± 4.2	19.0 ± 4.1	17.8 ± 5.1	15.9 ± 4.5	14.0 ± 3.8
LVSV/BSA (ml/m^2^)	43.2 ± 5.3	43.7 ± 5.5	42.1 ± 6.0	40.2 ± 7.5	40.9 ± 7.0
LVM/BSA (g/m^2^)	52.0 ± 5.9	52.6 ± 7.0	54.4 ± 7.0	57.8 ± 7.9	59.4 ± 6.8

*Abbreviations as in Table 2*.

**Table 5:** Absolute and indexed end-diastolic left ventricular wall thickness (LVWT) by age decile for men (*n* = 323).

	**21–30 (*n* = 41)**	**31–40 (*n* = 108)**	**41–50 (*n* = 95)**	**51–60 (*n* = 50)**	**61–70 (*n* = 29)**
**Absolute values**					
Global (mm)	7.7 ± 0.9	7.9 ± 1.0	8.2 ± 1.1	8.5 ± 1.0	8.7 ± 1.1
Basal (mm)	8.5 ± 0.8	8.7 ± 1.0	9.1 ± 1.2	9.3 ± 1.0	9.6 ± 1.3
Middle-cavity (mm)	7.6 ± 0.9	7.8 ± 1.0	8.0 ± 1.2	8.4 ± 1.0	8.5 ± 1.1
Apical (mm)	6.8 ± 1.1	7.0 ± 1.2	7.2 ± 1.3	7.7 ± 1.1	7.6 ± 0.9
**Indexed to BSA**					
Global (mm/m^2^)	4.3 ± 0.5	4.4 ± 0.5	4.5 ± 0.6	4.7 ± 0.6	4.9 ± 0.6
Basal (mm/m^2^)	4.7 ± 0.4	4.8 ± 0.5	5.0 ± 0.6	5.1 ± 0.5	5.5 ± 0.7
Middle-cavity (mm/m^2^)	4.2 ± 0.5	4.3 ± 0.5	4.4 ± 0.6	4.6 ± 0.6	4.8 ± 0.6
Apical (mm/m^2^)	3.7 ± 0.6	3.9 ± 0.7	3.9 ± 0.7	4.2 ± 0.7	4.3 ± 0.5

*BSA, body surface area*.

**Table 6:** Absolute and indexed end-diastolic LVWT by age decile for women (*n* = 227).

	**21–30 (*n* = 40)**	**31–40 (*n* = 60)**	**41–50 (*n* = 61)**	**51–60 (*n* = 39)**	**61–70 (*n* = 27)**
**Absolute values**					
Global (mm)	6.0 ± 0.6	6.3 ± 0.8	6.7 ± 0.8	7.2 ± 0.8	7.7 ± 1.0
Basal (mm)	6.5 ± 0.6	6.8 ± 0.9	7.3 ± 0.9	7.8 ± 0.9	8.4 ± 1.2
Middle-cavity (mm)	6.0 ± 0.6	6.1 ± 0.9	6.6 ± 0.8	7.1 ± 0.9	7.5 ± 0.9
Apical (mm)	5.2 ± 0.8	5.6 ± 1.4	5.8 ± 0.9	6.4 ± 0.9	6.8 ± 1.0
**Indexed to BSA**					
Global (mm/m^2^)	3.9 ± 0.5	4.0 ± 0.5	4.2 ± 0.5	4.6 ± 0.6	4.8 ± 0.5
Basal (mm/m^2^)	4.3 ± 0.5	4.4 ± 0.5	4.6 ± 0.5	5.0 ± 0.6	5.3 ± 0.6
Middle-cavity (mm/m^2^)	3.9 ± 0.5	3.9 ± 0.5	4.2 ± 0.5	4.5 ± 0.6	4.7 ± 0.5
Apical (mm/m^2^)	3.4 ± 0.6	3.6 ± 1.0	3.6 ± 0.5	4.0 ± 0.6	4.3 ± 0.5

*BSA, body surface area*.

**Table 7:** Absolute and indexed RV parameters by age decile for men (*n* = 323).

	**21–30 (*n* = 41)**	**31–40 (*n* = 108)**	**41–50 (*n* = 95)**	**51–60 (*n* = 50)**	**61–70 (*n* = 29)**
**Absolute values**					
RVEDV (ml)	140.7 ± 26.5	129.5 ± 20.8	129.2 ± 25.6	119.3 ± 18.5	116.0 ± 19.8
RVESV (ml)	62.1 ± 16.1	54.7 ± 12.2	54.2 ± 13.7	48.0 ± 11.4	44.2 ± 9.6
RVSV (ml)	79.4 ± 13.5	74.8 ± 12.5	74.8 ± 14.7	71.3 ± 9.7	71.8 ± 13.8
RVEF (%)	56.5 ± 5.1	57.9 ± 5.2	58.2 ± 4.7	60.1 ± 4.9	61.9 ± 5.7
RVCO (L/min)	5.5 ± 1.3	4.9 ± 0.9	4.8 ± 0.9	4.7 ± 0.9	4.8 ± 1.1
**Indexed values**					
RVEDV/BSA (ml/m^2^)	78.0 ± 11.6	71.1 ± 10.3	70.7 ± 12.7	65.1 ± 9.0	65.6 ± 9.9
RVESV/BSA (ml/m^2^)	34.1 ± 7.5	30.0 ± 6.1	29.6 ± 6.9	26.1 ± 5.6	25.0 ± 5.1
RVSV/BSA (ml/m^2^)	43.9 ± 6.3	41.1 ± 6.6	41.0 ± 7.4	38.9 ± 5.0	39.8 ± 8.3

*Abbreviations as in Table 2*.

**Table 8:** Absolute and indexed RV parameters by age decile for women (*n* = 227).

	**21–30 (*n* = 40)**	**31–40 (*n* = 60)**	**41–50 (*n* = 61)**	**51–60 (*n* = 39)**	**61–70 (*n* = 27)**
**Absolute values**					
RVEDV (ml)	107.0 ± 15.3	105.0 ± 13.4	102.2 ± 16.1	95.2 ± 22.1	94.6 ± 16.2
RVESV (ml)	41.8 ± 8.7	38.7 ± 8.0	37.1 ± 8.1	32.9 ± 8.7	30.3 ± 7.2
RVSV (ml)	65.3 ± 9.9	66.3 ± 8.6	65.2 ± 11.5	62.3 ± 14.7	64.3 ± 10.7
RVEF (%)	61.1 ± 5.0	63.3 ± 4.8	63.8 ± 5.4	65.5 ± 4.2	68.2 ± 4.6
RVCO (L/min)	4.5 ± 0.9	4.5 ± 0.7	4.3 ± 1.0	4.2 ± 1.0	4.4 ± 0.8
**Indexed values**					
RVEDV/BSA (ml/m^2^)	69.5 ± 7.4	66.6 ± 11.2	64.3 ± 9.5	59.8 ± 11.5	59.6 ± 9.8
RVESV/BSA (ml/m^2^)	27.1 ± 5.0	24.9 ± 4.9	23.3 ± 5.0	20.6 ± 4.7	19.1 ± 4.5
RVSV/BSA (ml/m^2^)	42.4 ± 5.0	42.7 ± 5.0	41.0 ± 6.7	39.2 ± 7.8	40.6 ± 6.6

*Abbreviations as in Table 2*.

### Reproducibility

**Figure 5:**
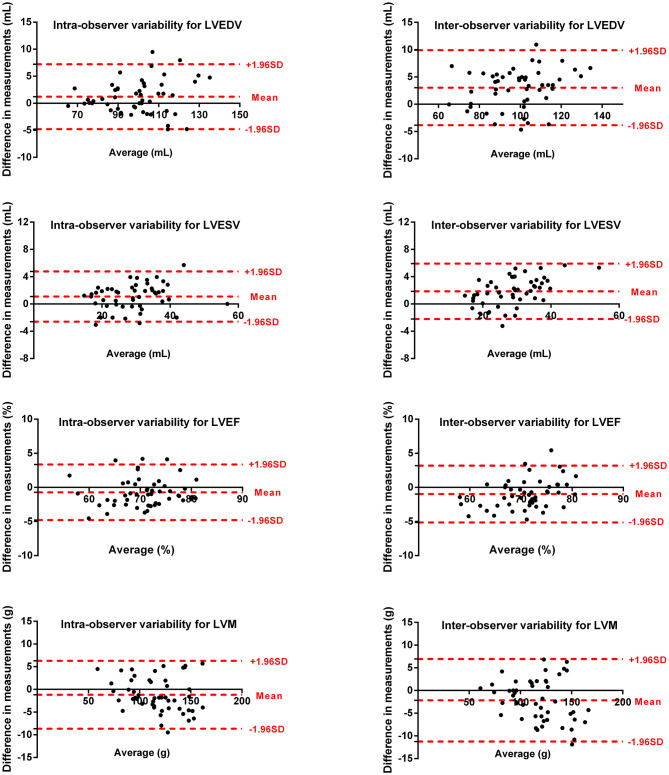
Bland–Altman plots for inter-observer and intra-observer reproducibility of LVEDV, LVESV, LVEF, and LVM.

**Figure 6:**
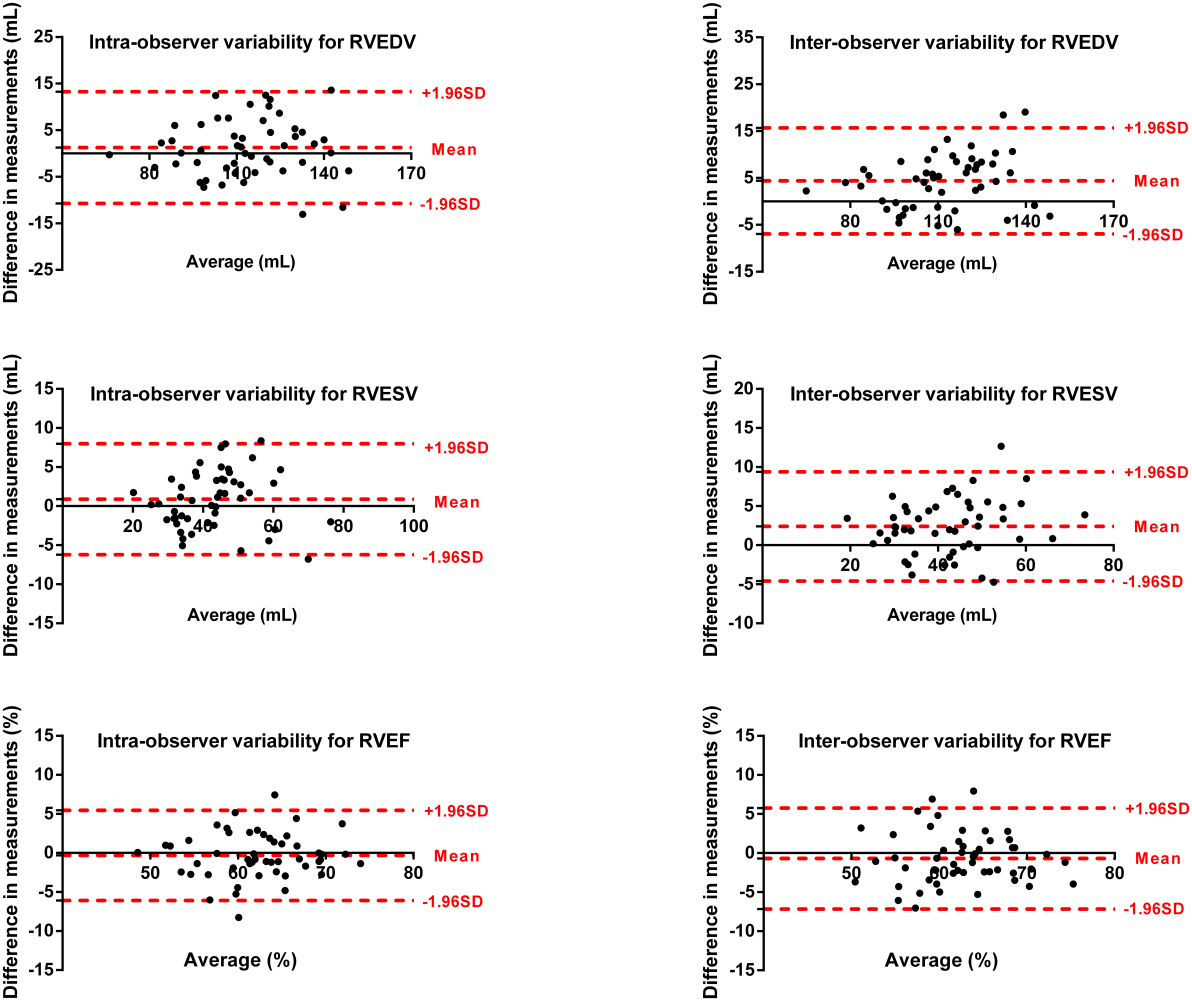
Bland–Altman plots for inter-observer and intra-observer reproducibility of RVEDV, RVESV, and RVEF.

Intra- and inter-observer variability for LV and RV measurements are shown in Figures 5, 6. The results showed excellent intra- and inter-observer agreements of biventricular measurements.

## Discussion

In this CMR study, we systematically established age-, sex-, and body size-specific normal values of biventricular morphology (volumes, mass, and wall thickness) and systolic function (EF, SV, and CO) based on a large sample of validated healthy Chinese adults over a wide age range. In addition, we further confirmed that biventricular structure and function were significantly associated with age and sex.

Several multi-ethnic studies confirmed that East Asians have a smaller ventricular chamber size than Westerners (9, 10). Similarly, we note that biventricular volumes of Chinese adults were significantly smaller than those of Caucasians, even after BSA normalization (19–21). In addition, compared with recently reported CMR reference ranges based on a systematic review of literatures on Westerners (22), we found that when using the same method of cavity delineation, the biventricular EDV and ESV for both male and female Chinese adults were smaller, while EF was slightly greater. Overall, all these observations suggest the great necessity of establishing cardiac reference values of different races or ethnicities. This study has some advantages over the previously reported CMR reference values for healthy Chinese adults (11–13). First, to our knowledge, this is the first CMR study to systematically provide age- and sex-specific normative values of global and segmental end-diastolic LVWT for Chinese adults, which may contribute to the accurate clinical diagnosis of various common cardiovascular diseases, such as cardiomyopathy. Second, the subjects included were confirmed healthy by rigorous whole-body health screening, and the sample size was significantly larger than that of similar previous CMR studies: Li et al. (11) (*n* = 90), Le et al. (12) (*n* = 180), and Lei et al. (13) (*n* = 120). Finally, the subjects included are of a wide age range (21–70 years) and were further stratified by sex and age deciles, so the obtained reference values have good clinical practicality. In comparison, the study of Li et al. (11) included only middle-aged adults aged 40–65 years, and the subjects were not further grouped by sex. Besides, although the subjects studied by Lei et al. (13) had a large age span (23–83 years), the cardiac reference values were divided into only two age groups (≥60 and <60 years).

It has been confirmed that the measurements of the LV volumes and systolic function could be significantly affected by the management of TPM (23, 24). However, there is still no uniform convention on the inclusion of TPM in the myocardium in terms of the LV chamber quantification. According to the most recent standard image interpretation and post-processing in CMR (17), TPM belongs to myocardial tissue and, thus, ideally should be included with the myocardium as part of the LVM. At present, due to the substantially improved blood–tissue contrast and higher spatial resolution of SSFP sequence, the commercially available, threshold-based segmentation technique could distinguish the boundary between blood pool and TPM with higher accuracy and efficiency and, thus, has been widely used in the clinical setting (25–27). Different from previous CMR studies on Chinese adults (11–13), we applied the current advanced threshold-based segmentation method with the inclusion of TPM in the myocardium, which may well account for the relatively smaller LV volumes, and relatively larger LVM and LVEF in this study.

In accordance with most previous studies (8, 11–13, 28), we confirmed that biventricular volumes, LVWT, and LVM in men were all greater than those in women. However, regarding the gender difference in indexed ventricular volumes, results are not always consistent. Specifically, after normalized by BSA, most studies reported that the ventricular volumes for men were comparable (11, 29) or significantly larger compared with those for women (13, 28, 30). We observed that BSA-indexed biventricular volumes including LVESV, RVEDV, and RVESV were all still greater in men, except for LVEDV. Besides, as the most commonly used indicator of cardiac systolic function, an early angiographic study from Buonanno et al. found a mean value for LVEF of 74% for women, significantly higher than the mean value of 67% for men (31). Similarly, we again observed that LVEF was significantly greater in women, which was consistent with several recent large-scale CMR or echocardiographic studies (19, 32, 33). A recent commentary by Kerkhof et al. demonstrated that EF increases disproportionally at smaller ESV (i) (34), which may well explain the larger LVEF in women. However, some studies demonstrated no significant gender difference in LVEF (11, 30). The authors consider that the ethnic differences, varied tracing methods, and demographic characteristics may collectively account for these discrepancies.

We confirmed that aging was associated with decreasing biventricular volumes and increasing end-diastolic LVWT, which was parallel to most previous reports (10–12, 21, 35). However, it is still controversial regarding the association of biventricular EF with aging. For example, a CMR study by Lei et al. (13) demonstrated that there was no significant correlation between EF and age. In contrast, we confirmed that both LVEF and RVEF were increasing with age, which was consistent with several previous studies (12, 20, 21, 36). In addition, the relationship between physiological aging and LVM has long been quite controversial. For instance, Petersen et al. (19) showed that LVM did not change significantly with age in either gender, and Cheng et al. (37) indicated that LVM was decreasing with age in both sexes. In the present study, we observed that there was a sexual dimorphism of association between age and LVM, evidenced by a significantly increased LVM with aging only for women, but not for men. The observed sex-specific association of aging with LVM agreed with some previous longitudinal echocardiographic studies (38, 39). The mechanism of gender differences in the effect of aging on LV remodeling is not clear. Hayward et al. (40) speculated that the normal decline in sex hormones with age has contrary effects on LVM in both sexes, which may well explain the gender difference in the association of LVM with aging. Consistent with our findings, several previous studies have demonstrated that aging was associated with the LV concentric remodeling in clinically healthy subjects (37, 41). Moreover, the present study further observed a greater age-related increase in LVM/LVEDV ratio in women, suggesting that women experienced more pronounced LV concentric remodeling with advancing age, which may be related to the fundamental gender differences in the myocardial response to chronic load alteration (42).

### Study Limitations

Some limitations should be acknowledged in this study. First, although we included a large sample of subjects with a broad age range, normative biventricular values of adults over 70 years old were still not covered due to the small number of the elderly population. Second, reference values of other cardiac functional parameters such as strain and strain rate were not included in the present study. Third, although we have tried to exclude subjects with cardiovascular diseases through strict exclusion criteria, techniques for diagnosing ischemic heart diseases such as late gadolinium enhancement imaging or coronary angiography were not performed in the present study. Finally, the associations of age with ventricular structure and function are based on the cross-sectional study, and follow-up studies are necessary in the future.

## Conclusions

In conclusion, we systematically provide age-, sex-, and body size-specific CMR reference values of biventricular morphology and function derived from a large sample of healthy Chinese adults. Biventricular structure and function are significantly associated with age and sex. The present study may be used as a reference standard for the diagnosis, risk stratification, and prognosis evaluation of cardiovascular disease in clinical research and practice.

## Data Availability Statement

The original contributions presented in the study are included in the article/supplementary material, further inquiries can be directed to the corresponding author/s.

## Ethics Statement

The studies involving human participants were reviewed and approved by Chinese Ethics Committee. The Ethics Committee waived the requirement of written informed consent for participation.

## Author Contributions

ZZhan contributed to imaging analysis, study design, and drafting of manuscript. QM and YG contributed to imaging analysis and statistical analysis. LC and CZ contributed to study supervision. ZZhao, JZ, and LZ organized the database. ML, GP, and KL contributed to study design and manuscript revision. All authors approved the final manuscript.

## Conflict of Interest

The authors declare that the research was conducted in the absence of any commercial or financial relationships that could be construed as a potential conflict of interest.
